# Fecal Evidence of Metal and Metalloid Exposure in a Free-Ranging Juvenile Southern Elephant Seal (*Mirounga leonina*) From the Brazilian Coast

**DOI:** 10.1007/s12011-026-05216-0

**Published:** 2026-07-13

**Authors:** Leila Soledade Lemos, Salvatore Siciliano, Rachel Ann Hauser-Davis

**Affiliations:** 1 Institute of Environment, Florida International University, North Miami, FL USA; 2 Departamento de Ciências Biológicas, Fundação Oswaldo Cruz (Fiocruz) Escola Nacional de Saúde Pública, Rio de Janeiro, RJ Brazil; 3 Fundação Oswaldo Cruz (Fiocruz), Instituto Oswaldo Cruz, Laboratório de Avaliação e Promoção da Saúde Ambiental, Rio de Janeiro, RJ Brazil

**Keywords:** Biomonitoring, E-waste, Marine mammal, Non-invasive matrix, Technology-related elements

## Abstract

Southern elephant seals are highly mobile marine predators that can reflect environmental exposure to chemical elements through their diet and foraging ecology. However, information on elemental concentrations in elephant seal feces remains scarce, particularly outside Antarctic regions. In this study, the concentrations of several metals and metalloids, including rarely studied technology-critical elements, were determined in fecal samples from a juvenile male southern elephant seal (*Mirounga leonina*) from along the southwestern Atlantic coast of Brazil. Elemental analyses revealed the predominance of biologically essential elements such as Ca and Fe, whereas toxic elements generally occurred at lower concentrations. Rare earth elements, as well as Li, Rb, and Ti were also detected, consistent with recent dietary exposure. Comparisons with previous Antarctic studies indicated overall similar or lower concentrations for most elements, although higher concentrations of As, Be, Cs, Cu, Rb, and U were observed herein. These differences may be associated with regional environmental conditions as well as ontogenetic variations in diet between juvenile and adult individuals. This study provides the first baseline data on elemental concentrations in southern elephant seal feces from the southwestern Atlantic and highlights the usefulness of feces as a non-invasive matrix for monitoring recent environmental exposure in marine ecosystems.

**Supplementary Information:**

The online version contains supplementary material available at https://doi.org/10.1007/s12011-026-05216-0.

## Introduction

 The southern elephant seal (*Mirounga leonina*) is the largest member of the Phocidae family and one of the most widely distributed pinnipeds in the Southern Hemisphere [43]. These seals breed and molt on sub-Antarctic islands such as South Georgia, Kerguelen Islands, Heard Island, Macquarie Island, and Peninsula Valdés in Argentina [23, 28] and forage across vast distances in the Southern Ocean [16]. Although their core distribution is polar and subpolar, occasional vagrant individuals are recorded far outside their typical range, including along the southeastern coast of Brazil [10].

As top predators with high trophic levels and long lifespans, elephant seals are susceptible to the accumulation of environmental contaminants, including metals and metalloids [1]. Southern elephant seals primarily feed on mesopelagic fish, squid, and occasionally krill, foraging at great depths across oceanic regions [2, 8], making dietary intake a major pathway for contaminant exposure. Metals and metalloids enter marine food webs through natural and anthropogenic sources and may accumulate in apex predators due to bioaccumulation and biomagnification processes [13], supporting the use of elephant seals as indicators of marine contaminant exposure.

Despite their ecological significance, few studies have evaluated contaminant loads in *M. leonina*, and existing work has focused primarily on mercury (Hg), for example, Cossaboon et al. [7], who analyzed Hg concentrations in the fur of adult northern elephant seals from California (*Mirounga angustirostris*); Peterson et al. [34], who evaluated the relationships between mercury concentrations in blood, muscle, and hair of female and male northern elephant seals, also in California; and Kehrig et al., and [20], who examined Hg in lanugo (natal fur) from southern elephant seal pups in Antarctica. These studies provide important insights but remain limited in scope, particularly with respect to other elements and alternative biological matrices. Traditional monitoring of metal and metalloid contamination in marine mammals often relies on internal tissues such as the liver, kidney, and muscle [15, 24, 26, 29], which require animal sacrifice and therefore pose ethical and logistical challenges, especially for protected or migratory species [44]. Non-lethal approaches have been developed using keratinized tissues such as fur, which generally reflect longer-term exposure and provide an alternative means of assessing contaminant burdens [34] and, more recently, fecal samples have emerged as an additional non-invasive matrix for environmental monitoring [37]. In this sense, as fecal matter reflects recent dietary intake and excretion processes, it provides a complementary proxy for assessing contaminant exposure via ingestion [25]. Despite these advantages, the use of fecal analysis to assess exposure to metals and metalloids in elephant seals remains largely unexplored.

To the best of our knowledge, only three studies have assessed metal loads in elephant seal fecal samples, all in Antarctic populations [5, 35, 47]. While these studies provide valuable baseline information on elemental exposure in southern elephant seals, fecal-based assessments remain rare and have not yet been explored in other regions or under different environmental contexts. This study aimed to quantify, for the first time, a suite of metals and metalloids in a fecal sample of a southern elephant seal collected in Rio de Janeiro, Brazil. The feasibility of feces as a non-invasive matrix for contaminant monitoring in this species was evaluated by assessing the presence and variability of several elements, selected to encompass (i) biologically essential elements involved in physiological and metabolic processes (e.g., Ca, Fe, Zn, Cu, Se), (ii) toxic elements of established ecotoxicological concern in marine food webs (e.g., As, Cd, Hg, Pb), and (iii) emerging contaminants, including rare earth elements (REEs) and technology-critical elements such as lithium (Li), rubidium (Rb), and titanium (Ti). The environmental occurrence emerging contaminants, in particular, has increased due to technological and industrial applications and their association with electronic waste and increasing environmental release. This broad elemental screening was intended to provide an initial characterization of both conventional and emerging contaminant exposure in southern elephant seals, with potential relevance for marine conservation and monitoring efforts aligned with the United Nations Sustainable Development Goals.

## Methods

**Fig. 1:**
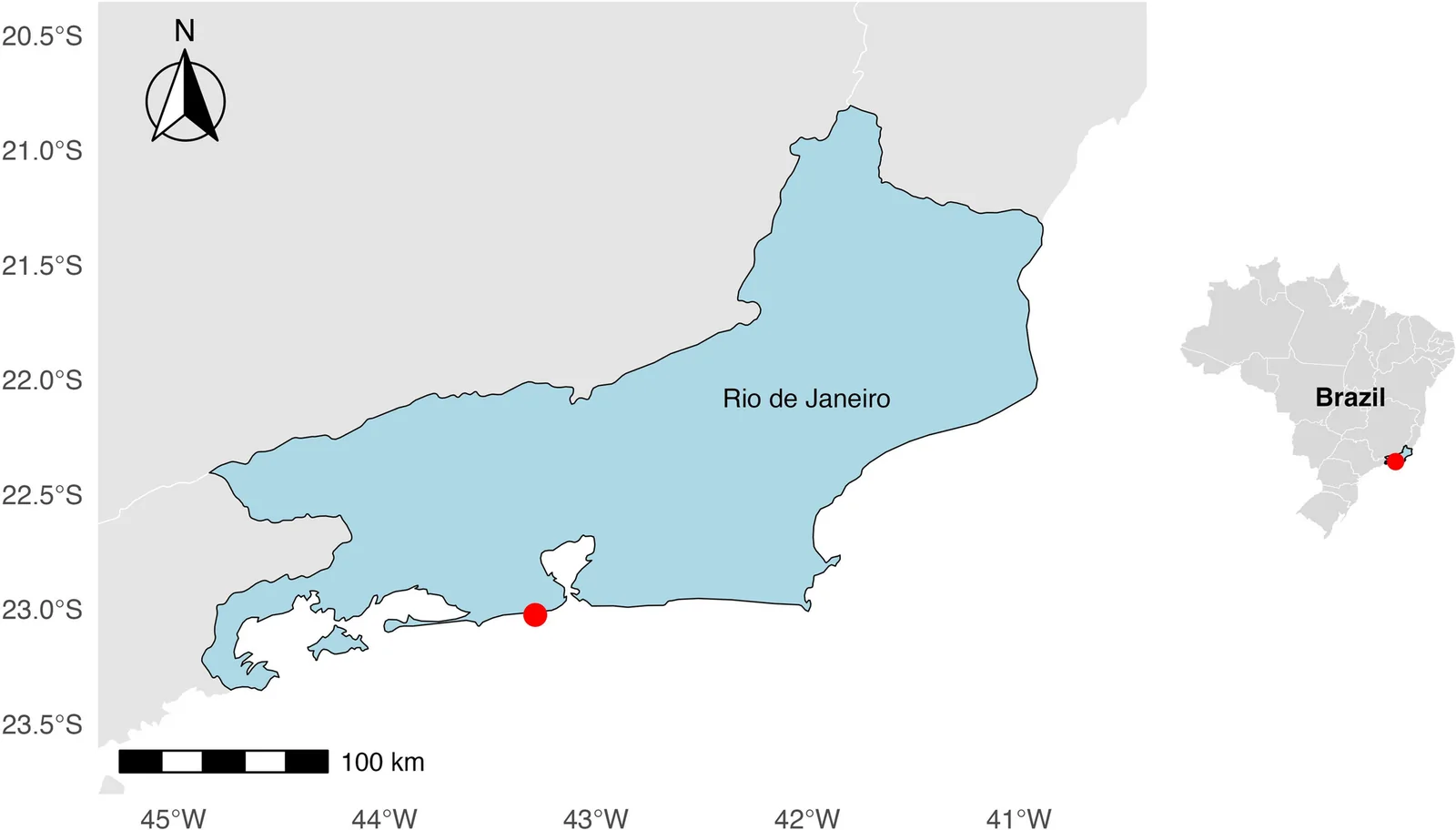
Geographic location of the sampling site in Rio de Janeiro state, Brazil. The inset map shows the broader location within the country. The red point denotes the site where a fecal sample from a juvenile elephant seal (*Mirounga leonina*) was collected for element quantification

On 3 March 2020, a juvenile male southern elephant seal (~ 1.3 m in length) was recorded resting on Barra da Tijuca Beach, Rio de Janeiro, southeastern Brazil (Fig. 1). The sampled individual was likely a young-of-the-year or recently weaned individual, based on morphometric characteristics described for southern elephant seals. Previous reports indicate that juvenile and subadult southern elephant seals occasionally occur as vagrants along the southeastern Brazilian coast, likely transported by oceanographic conditions and long-distance dispersal from subantarctic breeding colonies (e.g., Patagonia or South Georgia) [39].

During its stay, the animal defecated, and the fecal sample was immediately collected using a sterile polypropylene tube. This unexpected encounter provided a rare opportunity to analyze metals and metalloids in feces from a free-ranging individual.

The sample was weighed (~ 100 mg) and mixed with 1 mL of subdistilled nitric acid (67% v/v, Sigma-Aldrich, São Paulo), left overnight, and heated at 100 °C for 5 h the following day. After cooling, the volume was adjusted to 10 mL and diluted with ultrapure water (resistivity > 18 MΩ cm) for subsequent analysis by inductively coupled plasma mass spectrometry (ICP − MS).

Quality assurance was ensured using procedural blanks and certified reference materials (BCR 668 – Mussel Tissue and BB442 – Fish Muscle) used in this study. The ICP-MS conditions were optimized using multi-element calibration curves prepared from appropriate dilutions of a mixed standard solution (Merck IV). All calibration curves showed correlation coefficients greater than 0.995, and all certified reference material recoveries ranged from 70% to 114%, adequate for this type of assessment. Minor deviations from the ideal recovery range (± 10%) were attributed to matrix-related effects and inherent analytical variability associated with ICP-MS measurements of complex biological tissues. Nevertheless, all recoveries fell within acceptable limits for trace-element determinations and were considered adequate for the present application. A total of 56 elements were quantified using ICP-MS. From these, nine were below the limit of quantification (Ag, Au, Bi, Ge, In, Pt, Re, Ta, and W; Table S2).

## Results and Discussion

The elemental levels found herein were: Ca > Si > Fe > Sr > Br > Zn > Ti^#^ > Mn > Cu > B > I > Ba > Ce* > Ni > V > Se > Pb > As > La* > Li^#^ > Rb^#^ > Sc* > Y* > Cd > Pr* > Co > Ga > Sm* > Zr > Gd* > U > Nb > Mo > Pd > Hg > Er* > Cs > Yb* > Eu* > Be > Tb* > Sn > Sb > Ho* > Tl > Lu* > Tm* (Table 1; asterisks represent REEs and # represent Technology Critical Elements). Overall, the elemental profile was dominated by major essential elements such as Ca and Fe, which are consistent with physiological processes and dietary intake, particularly from marine prey such as fish and squid, which are known to be rich in microminerals essential for metabolic and structural functions [22, 36]. Silicon also displayed a comparatively high concentration and likely reflects trophic transfer of biogenic silica from diatom-based food webs (e.g., diatoms > zooplankton > fish and squid > elephant seal), as well as potential contributions from environmental particulate matter [42]. In contrast, toxic elements such as As, Cd, Hg and Pb were detected at comparatively low concentrations, likely indicating limited recent exposure [14].

**Table 1:** Concentrations of metals and metalloids in fecal samples of elephant seals (*Mirounga leonina*) reported in this study (*N* = 1; juvenile male from Southeastern Brazil; reported in wet weight [ww]; and converted to dry weight [dw] considering the moisture content of 58% [21] and previous studies conducted in Antarctica ([35]; *N* = 4; sex and age group not specified; and Celis et al., and [5]; *N* = 3; adults). Values are presented as mean ± standard deviation with range in parentheses (µg g⁻¹). “–” indicates not reported, “<” indicates below the Limit of Quantification, and “ND” indicates not detected in other studies

Element	This Study [ww]	This study [dw]	Reindl et al. [35] [dw]	Celis et al. [5] [dw]
Ag	< 0.00154	< 0.00367	-	0.08 ± 0.014 (0.07–0.09)
As	0.199	0.475	-	0.153 ± 0.123 (0.07–0.24)
Au	< 0.00091	< 0.00217	-	0.0003 ± 0.0004 (0.00004–0.0006)
B	0.515	1.226	-	-
Ba	0.412	0.982	-	23.21 ± 0.76 (22.67–23.75)
Be	0.003	0.008	-	ND
Bi	< 0.00057	< 0.00136	-	0.0010 ± 0.0007 (0.0005–0.0015)
Br	13.519	32.189	-	-
Ca	5314	12,653	-	-
Cd	0.047	0.112	-	0.342 ± 0.489 (0.05–0.91)
Ce	0.337	0.803	22.9 ± 10.9 (12.64–34.4)	-
Co	0.029	0.070	-	8.09 ± 6.87 (0.70–14.29)
Cr	-	-	-	1.74 ± 1.35 (0.19–2.54)
Cs	0.005	0.012	-	0.0028 ± 0.0002 (0.0027–0.003)
Cu	0.825	1.965	-	0.183
Dy	-	-	2.27 ± 1.17 (1.40–3.60)	-
Er	0.005	0.013	1.15 ± 0.576 (0.728–1.80)	-
Eu	0.003	0.008	0.718 ± 0.273 (0.539–1.03)	-
Fe	93.471	222.5	-	3184.4 ± 4059.6 (313.8–6055.0)
Ga	0.027	0.064	-	4.57 ± 1.86 (3.45–6.71)
Gd	0.022	0.052	4.22 ± 2.15 (2.40–6.60)	-
Ge	< 0.00247	< 0.00588	-	0.015 ± 0.010 (0.01–0.03)
Hf	-	-	-	0.141 ± 0.171 (0.02–0.26)
Hg	0.005	0.013	-	-
HgII	-	-	-	125.6 ± 91.4 (64.6–230.7)
MeHg	-	-	-	90.8 ± 82.4 (42.6–185.9)
Ho	0.002	0.004	0.422 ± 0.219 (0.263–0.671)	-
I	0.444	1.058	-	-
In	< 0.00046	< 0.00110	-	0.0014 ± 0.0003 (0.0012–0.0016)
K	-	-	-	-
La	0.198	0.470	9.54 ± 4.75 (5.15–14.59)	-
Li*	0.165	0.393	-	1.23 ± 1.06 (0.01–1.89)
Lu	0.001	0.002	0.149 ± 0.060 (0.102–0.216)	-
Mn	1.253	2.984	-	211.6 ± 239.6 (42.1–381.02)
Mo	0.012	0.029	-	0.104 ± 0.117 (0.01–0.24)
Nb	0.013	0.030	-	0.02 ± 0.028 (0.0034–0.05)
Nd	-	-	14.6 ± 7.35 (8.02–22.5)	-
Ni	0.333	0.794	-	3.25 ± 2.39 (0.68–5.41)
Pb	0.208	0.494	-	2.17 ± 0.57 (1.34–3.22)
Pd	0.011	0.025	-	-
Pr	0.041	0.098	3.30 ± 1.62 (1.83–5.04)	-
Pt	< 0.00062	< 0.00148	-	-
Rb*	0.137	0.327	-	0.057 ± 0.011 (0.049–0.065)
Re	< 0.00029	< 0.00069	-	0.0004 ± 0.0004 (0.0001–0.0007)
Sb	0.002	0.004	-	0.004 ± 0.005 (0.0005–0.008)
Sc	0.058	0.139	8.60 ± 4.44 (3.53–11.8)	-
Se	0.271	0.645	-	11.95
Si	292.754	697.0	-	-
Sm	0.027	0.063	3.28 ± 1.65 (1.86–5.09)	-
Sn	0.002	0.006	-	0.014 ± 0.018 (0.001–0.027)
Sr	37.347	88.92	-	116.24 ± 7.01 (109.23–123.25)
Ta	< 0.00052	< 0.00124	-	0.0029 ± 0.0007 (0.0021–0.0034)
Tb	0.003	0.007	0.475 ± 0.243 (0.283–0.748)	-
Th	-	-	-	0.073 ± 0.057 (0.03–0.11)
Ti*	2.644	6.294	-	42.6 ± 54.9 (3.74–81.52)
Tl	0.001	0.003	-	0.432 ± 0.154 (0.32–0.54)
Tm	0.001	0.002	0.158 ± 0.075 (0.103–0.243)	-
U	0.020	0.047	-	0.0068 ± 0.002 (0.005–0.008)
V	0.302	0.719	-	36.27 ± 8.49 (30.26–42.27)
Y	0.056	0.134	10.9 ± 6.10 (6.38–17.8)	-
Yb	0.004	0.010	0.940 ± 0.375 (0.648–1.36)	-
W	< 0.00100	< 0.00238	-	0.003 ± 0.005 (0.0003–0.01)
Zn	9.096	21.658	-	978.8 ± 1506.4 (99.8–2718.2)
Zr	0.024	0.057	-	4.11 ± 4.79 (0.72–7.5)

Out of the 17 existing REEs, 14 were analyzed herein, all of which were detected, indicating their widespread presence. Rare earth elements are widely distributed in the Earth’s crust, where they are initially concentrated through magmatic, tectonic, and hydrothermal processes and subsequently released into the environment through weathering of rocks and soils [9]. They are transported to marine systems via riverine input, atmospheric deposition, and sediment resuspension [33]. In addition to these natural sources, REEs have increasing anthropogenic relevance due to their extensive use in modern technologies, including electronics, renewable energy systems, catalysts, and medical applications, which can contribute to their release into aquatic environments through industrial effluents and waste disposal [31]. Exposure to REEs has been associated with several health effects, including severe respiratory issues, genetic damage, systemic toxicity, oxidative stress, cell death in human tissue, elevated risks of hypertension, renal dysfunction, liver damage, increased likelihood of fetal neural tube defects, reduced fertility, and male sterility [3, 18, 45].

Similarly, Li, Rb, and Ti are naturally occurring elements whose environmental concentrations have been increasingly influenced by technology-related human activities. These elements are widely used in industrial and technological applications, including batteries, nuclear medicine, electronics, alloys, and photo cells [19, 30, 46] Although historically overlooked in marine ecotoxicology, Li is increasingly recognized as an emerging contaminant due to its rising environmental concentrations and potential endocrine-disrupting and toxic effects on aquatic organisms, including behavioral alterations, oxidative stress, and reproductive impairment [6, 17]. As for Rb, it has limited biological function but potential incorporation into food webs and biomagnification due to its chemical similarity to potassium [4]. Excessive exposure to Rb may lead to weight loss, loss of coordination, skin ulcers, and extreme nervousness [30]. Titanium, in turn, is generally considered to have low bioavailability and relatively low toxicity due to its high chemical stability and low solubility [27]. Nevertheless, certain titanium-based compounds, particularly titanium dioxide (TiO₂) nanoparticles, which have increased significantly in marine environments due to their high use in personal care products, have raised environmental concerns because they may induce oxidative stress, cellular and genetic damage, and altered physiological responses in marine organisms, especially under chronic exposure conditions [11, 40]. Titanium has also been reported to accumulate in organs such as the heart, lungs, and spleen; to cause lower reproduction rates and early deaths in male rats [38].

Considering the other few studies assessing metal loads in elephant seal fecal samples in Antarctica [5, 35, 47], the concentrations found herein were overall similar or lower, except for As, Be, Cs, Cu, Rb, and U (Table 1). These patterns likely reflect a combination of dietary exposure and regional geochemical background, although the limited number of comparative datasets for elephant seal feces constrains broader generalizations. This also represents the first effort to quantify the elements Br, Ca, I, Pd, and Si, thus establishing preliminary baseline concentrations for these elements in southern elephant seal feces.

The As, Be, Cs, Cu, Rb, and U concentrations found herein were higher than those reported by Celis et al. [5].This difference may be influenced by ontogenetic variation in diet, as juvenile and adult elephant seals exhibit distinct distribution, foraging strategies and prey compositions, which can affect their exposure to elements via trophic transfer [32, 41]. Common prey of juveniles (1–3 years old) includes squid species such as *Alluroteuthis antarcticus*, *Histioteuthis eltaninae*, and *Slosarczykovia circumantarcticus* [12]. Adults, in turn, have a more varied diet, including bigger squids, fish, ascidians, crustaceans, and other invertebrates [32, 41].

This study has certain limitations that should be considered when interpreting the findings. First, the results are based on a single fecal sample from one juvenile southern elephant seal, which precludes broader inferences regarding population-level elemental exposure, temporal variability, sex- or age-related differences, and individual variation in diet or physiology. Therefore, the concentrations reported herein should be interpreted as preliminary and exploratory rather than representative of southern elephant seals in the southwestern Atlantic. Second, although feces provide a valuable non-invasive matrix for contaminant assessment, they primarily reflect recent dietary exposure and excretion rather than long-term body burdens or tissue accumulation, especially considering the relatively rapid gut passage time reported for elephant seals (~ 13 h; [21]). Consequently, the detected elemental profile likely reflects short-term exposure linked to recent foraging activity and may not fully represent cumulative contaminant uptake over longer temporal scales. Methodological limitations should also be acknowledged. Comparisons with previous studies are constrained by differences in sample size, analytical approaches, age classes, and geographic regions, which may influence apparent concentration differences among studies. Additionally, toxicokinetic information in marine mammals for several emerging contaminants, including REEs and technology-critical elements, remains limited, restricting interpretation regarding bioaccumulation, physiological handling, and toxicological significance. Future studies incorporating larger sample sizes, multiple age classes, longitudinal sampling, and complementary tissues are needed to better characterize exposure pathways and ecological risks in elephant seals.

## Conclusions

This study provides the first comprehensive assessment of elemental concentrations in southern elephant seal feces in the southwestern Atlantic coast, revealing the widespread presence of major, technology-related, and rare earth elements. The elemental profile was dominated by biologically essential elements, while toxic elements occurred at generally lower concentrations, likely reflecting limited exposure in the sampled individual. The detection of REEs, Li, Rb, and Ti likely indicates recent dietary exposure to e-waste. Differences observed for certain elements relative to Antarctic studies may also reflect ontogenetic variation in diet, highlighting the importance of considering life stage when interpreting trace element profiles in highly mobile marine predators. These findings provide preliminary data on elemental concentrations in a southern elephant seal from the southwestern Atlantic, contributing an initial point of comparison for future monitoring and regional studies. Elephant seal feces are noted as a valuable non-invasive matrix for assessing recent environmental exposure and elemental transfer through marine food webs.

## Supplementary Information

Below is the link to the electronic supplementary material.

Supplementary material (DOCX 92.6 KB)

## Data Availability

Data will be made available upon request.
