# Supplementary material for: Fecal Evidence of Metal and Metalloid Exposure in a Free-Ranging Juvenile Southern Elephant Seal (Mirounga leonina) From the Brazilian Coast
Source: Biol Trace Elem Res. 2026 Jul 13;204(10):7702–10. doi: 10.1007/s12011-026-05216-0 (PMC13615982; doi:10.1007/s12011-026-05216-0)
Supplement: Supplementary file 1 [file 12011_2026_5216_MOESM1_ESM.docx]

**Supplementary Information**

**Table S1.** Certified, observed, and recovery (%) values for the Certified Reference Materials (BCR 668 – Mussel Tissue and BB442 – Fish Muscle) used in this study.

| **Element** | **BCR 668** | | |  | **BB422** | | |
| --- | --- | --- | --- | --- | --- | --- | --- |
|  | **Observed** | **Certified** | **Recovery (%)** | **Element** | **Observed** | **Certified** | **Recovery (%)** |
| **As** | 7.9 | 7.1 | 111 | As | 13.7 | 12.7 | 108 |
| **Ca** | - | - | - | Ca | 373 | 342 | 109 |
| **Cd** | 0.244 | 0.275 | 89 | Cd | 0.0060 | 0.0075 | 80 |
| **Ce** | 0.062 | 0.089 | 70 | Ce | - | - | - |
| **Co** | 0.31 | 0.31 | 101 | Co | - | - | - |
| **Cs** | 0.0097 | 0.0138 | 71 | Cs | - | - | - |
| **Cu** | - | - | - | Cu | 1.40 | 1.67 | 84 |
| **Er** | 0.0034 | 0.0045 | 75 | Er | - | - | - |
| **Fe** | 70.6 | 84.7 | 83 | Fe | 8.4 | 9.4 | 89 |
| **Gd** | 0.010 | 0.013 | 75 | Gd | - | - | - |
| **Hg** | - | - | - | Hg | 0.555 | 0.601 | 92 |
| **Ho** | 0.0014 | 0.0018 | 80 | Ho | - | - | - |
| **I** | - | - | - | I | 1.6 | 1.4 | 114 |
| **La** | 0.06 | 0.08 | 75 | La | - | - | - |
| **Lu** | 0.000314 | 0.000398 | 79 | Lu | - | - | - |
| **Mg** | - | - | - | Mg | 1099 | 1370 | 80 |
| **Mn** | - | - | - | Mn | 0.320 | 0.368 | 87 |
| **Mo** | 2.18 | 1.99 | 110 | Mo | - | - | - |
| **Pr** | 0.0090 | 0.0123 | 73 | Pr | - | - | - |
| **Se** | - | - | - | Se | 1.47 | 1.33 | 111 |
| **Sm** | 0.0082 | 0.0112 | 73 | Sm | - | - | - |
| **Tb** | 0.00130 | 0.00162 | 80 | Tb | - | - | - |
| **Tb** | 0.00036 | 0.00048 | 76 | Tb | - | - | - |
| **U** | 0.053 | 0.056 | 94 | U | - | - | - |
| **Y** | 0.042 | 0.059 | 71 | Y. | - | - | - |
| **Yb** | 0.0021 | 0.0028 | 74 | Yb | - | - | - |
| **Zn** | 54.1 | 70.7 | 77 | Zn | 13 | 16 | 79 |

**Table S2.** Limits of detection (LOD) and limits of quantification (LOQ) for the analyzed elements in the study sample. Values are expressed in μg g⁻¹.

| **Element** | **LOD** | **LOQ** | **Element** | **LOD** | **LOQ** |
| --- | --- | --- | --- | --- | --- |
| **Ag** | 0.00046 | 0.00154 | **Mo** | 0.00060 | 0.00200 |
| **As** | 0.00256 | 0.00854 | **Nb** | 0.00026 | 0.00085 |
| **Au** | 0.00027 | 0.00091 | **Ni** | 0.00651 | 0.02169 |
| **B** | 0.05589 | 0.18630 | **Pb** | 0.00054 | 0.00179 |
| **Ba** | 0.00324 | 0.01080 | **Pd** | 0.00054 | 0.00182 |
| **Be** | 0.00018 | 0.00060 | **Pr** | 0.00005 | 0.00017 |
| **Bi** | 0.00179 | 0.00057 | **Pt** | 0.00018 | 0.00062 |
| **Br** | 0.03885 | 0.12951 | **Rb** | 0.00027 | 0.00090 |
| **Ca** | 0.65987 | 2.19956 | **Re** | 0.00009 | 0.00029 |
| **Cd** | 0.00048 | 0.00161 | **Sb** | 0.00026 | 0.00088 |
| **Ce** | 0.00008 | 0.00028 | **Sc** | 0.02768 | 0.09227 |
| **Co** | 0.00019 | 0.00063 | **Se** | 0.00944 | 0.03148 |
| **Cs** | 0.00006 | 0.00019 | **Si** | 28.3353 | 94.4510 |
| **Cu** | 0.00156 | 0.00519 | **Sm** | 0.00017 | 0.00058 |
| **Er** | 0.00005 | 0.00018 | **Sn** | 0.00054 | 0.00181 |
| **Eu** | 0.00011 | 0.00037 | **Sr** | 0.00039 | 0.00132 |
| **Fe** | 0.07806 | 0.26019 | **Ta** | 0.00015 | 0.00052 |
| **Ga** | 0.00029 | 0.00097 | **Tb** | 0.00004 | 0.00013 |
| **Gd** | 0.00013 | 0.00045 | **Ti** | 0.00267 | 0.00891 |
| **Ge** | 0.00074 | 0.00247 | **Tl** | 0.00006 | 0.00020 |
| **Hg** | 0.00097 | 0.00322 | **Tm** | 0.00003 | 0.00012 |
| **Ho** | 0.00003 | 0.00012 | **U** | 0.00014 | 0.00047 |
| **I** | 0.00499 | 0.01663 | **V** | 0.00016 | 0.00055 |
| **In** | 0.00014 | 0.00046 | **W** | 0.00030 | 0.00100 |
| **La** | 0.00006 | 0.00020 | **Y** | 0.00010 | 0.00032 |
| **Li** | 0.00069 | 0.00230 | **Yb** | 0.00007 | 0.00025 |
| **Lu** | 0.00003 | 0.00009 | **Zn** | 0.01159 | 0.03863 |
| **Mn** | 0.00028 | 0.00095 | **Zr** | 0.00025 | 0.00083 |
